# Guidelines for ketamine use in clinical psychiatry practice

**DOI:** 10.1192/bjo.2024.62

**Published:** 2024-05-10

**Authors:** Luke A. Jelen, Rupert McShane, Allan H. Young

**Affiliations:** The Maudsley Hospital, London, UK; and Institute of Psychiatry, Psychology & Neuroscience, King's College London, UK; Department of Psychiatry, Oxford University, UK; and Oxfordshire ECT and Ketamine Service, Oxford, UK; Centre for Affective Disorders, Institute of Psychiatry, Psychology & Neuroscience, King's College London, UK

**Keywords:** Depressive disorders, antidepressants, ketamine, esketamine, guidelines

## Abstract

In this editorial, we emphasise the efficacy and challenges of using ketamine in treatment-resistant depression. We highlight the need for comprehensive evidence-based guidelines to manage the use of both licensed and off-licence ketamine formulations and discuss recent efforts by Beaglehole et al to develop ketamine guidelines in New Zealand. We finally advocate for national registries to monitor ketamine therapy, ensuring its responsible and effective use in the management of depression.

The efficacy of ketamine in the treatment of treatment-resistant depression (TRD) is well documented,^1,2^ and ketamine-based therapies offer new therapeutic hope for those that do not experience beneficial outcomes from conventional antidepressants. Consequently, there has been growing interest in incorporating the use of ketamine into psychiatry in diverse settings across the globe. However, the transition of ketamine treatments from research and specialist settings to routine clinical practice poses significant challenges, including concerns over potential misuse, dissociative effects and uncertainty regarding longer-term risks.

Ketamine is a racemic mixture composed of equal amounts of (S)- and (R)-ketamine (esketamine and arketamine).^3^ Although an esketamine nasal spray has been developed and licensed for use in TRD (in combination with a conventional antidepressant) in Europe and New Zealand, neither the National Institute for Health and Care Excellence nor Pharmac (respective government agencies that evaluate cost-effectiveness of new treatments) has approved its public use. For some, the licensing of esketamine for TRD remains a controversial decision, and this has been a topic of considerable debate.^4,5^ In the UK, although there has been some limited National Health Service use of nasal esketamine, approved on a named-patient basis, its availability is primarily confined to select, often costly, private providers. Owing to the risks of sedation, dissociation and misuse, in certain countries intranasal esketamine is only available through a restricted distribution system under a risk evaluation and mitigation strategy that specifies standards for healthcare settings, pharmacies and healthcare professionals administering the drug.

Meanwhile, the use of racemic ketamine for depression is increasing across public and private sectors as a cost-efficient off-label approach. Intravenous (i.v.) administration of ketamine is recognised as the gold standard for off-label use, with the best supporting evidence for efficacy in TRD.^2^ Other modes of administering ketamine including subcutaneous, intramuscular (i.m.), oral and sublingual are being explored but require further research to validate their relative safety and efficacy and to determine the optimal dosing regime in each case. Each administration route presents distinct advantages and challenges relating to bioavailability, effect duration, practicality and patient comfort. Importantly, none of these treatment modes for racemic ketamine has received regulatory approval for on-label use for any psychiatric indication. As a result, there are no formal surveillance data on safety and effectiveness.^6^ Therefore, there is a critical need to establish international expert consensus opinion, alongside comprehensive and clear guidelines to manage off-label use, including dosage recommendations for different administration routes and requisite monitoring practices.

To date, the major evidence-based guidelines for treating depressive disorders have either not mentioned^7^ or only briefly touched on ketamine, with no formal recommendation for its use in depression^8,9^ aside from that limited to specialist academic treatment centres.^10^ However, a key consensus paper from an international group of mood disorders experts provides a helpful synthesis with respect to the efficacy, safety and tolerability of ketamine and esketamine in TRD.^11^ This review of the evidence supports the rapid-onset efficacy (within 1–2 days) of esketamine and ketamine in TRD, which is best established for intranasal esketamine and i.v. ketamine routes. Conversely, there is rather limited evidence supporting the efficacy of oral, subcutaneous or i.m. ketamine in TRD. Intranasal esketamine has proven effective, safe and tolerable for up to 1 year in TRD, although the long-term effects of i.v. ketamine remain insufficiently studied.^12^ Both ketamine and esketamine give rise to safety concerns encompassing psychiatric (dissociation, psychotomimetic and increased suicidality), neurological/cognitive, genitourinary and hemodynamic effects that require monitoring. The Ketamine Side Effect Tool was been developed as one approach to systematically monitor and report ketamine-related side-effects.^13^ Considering safety concerns, the consensus view is that these compounds should be administered in environments with multidisciplinary personnel, including experts in mood disorder assessment. To aid clinicians and healthcare providers, a detailed discussion of the risks and practical recommendations for the use of oral, sublingual and nasal ketamine has been recently outlined.^14^

We welcome the efforts by Beaglehole et al^15^ to establish ketamine guidelines for use by adult specialist mental health services in New Zealand. A particularly novel aspect is that they seek to address the need for long-term treatment in a way that is scalable in a public health service. The primary identification of TRD patients for potential ketamine treatment provides a reasonable pathway for identifying those in need of intervention. The authors highlight a paradox in the clinical adoption of off-label ketamine for treating depression: clinicians’ hesitance to use it is perpetuated by a lack of first-hand experience. The underpinning published research, on its own, appears to have been insufficiently persuasive to overcome this hesitancy and risk aversion. To address this, the authors propose an approach beginning with i.m. administration to gauge patient response, followed by an oral regimen. It is reasonable to use parenteral administration as a test of responsiveness and then follow this with something more pragmatic, but, as described, the current evidence base is for i.v. not i.m.; i.m. may give a variable response that depends more on administration technique. Another challenge the authors highlight is establishing the requisite experience level for psychiatrists to prescribe ketamine. It is recommended that psychiatrists observe at least three i.m. administrations to become acquainted with the dissociative effects experienced by patients. The guidelines suggest a maximum treatment duration of 12 weeks. This duration is a balance between practicality – allowing sufficient time to evaluate clinical responses and reinforce benefits – and caution, as the authors were reluctant to endorse long-term treatment. However, this approach could introduce complications, especially as ketamine-responders may, like responders to esketamine nasal spray, be at a high risk of relapse following cessation of regular dosing. Finally, we agree with the authors about the necessity of diligent monitoring in ketamine therapy clinics, with particular emphasis on assessing mood and cognitive function, which are crucial indicators of a patient's response to ketamine treatment. However, although the guidelines promote oral administration as a strategy to enhance treatment accessibility, this approach potentially increases the risks associated with overuse and potential misuse. Therefore, it underscores the need for more stringent oversight across any healthcare facilities offering ketamine therapies.

To address safety concerns and monitor the effectiveness of ketamine treatments, we believe it is crucial to establish mandatory national registers encompassing all individuals receiving these treatments, whether licensed or unlicensed. Such registries would address risk mitigation, facilitate pharmacovigilance and enable tracking of patient outcomes across diagnoses, routes and doses. In every state of the USA and in the Australian states of Queensland, Victoria and South Australia, Prescription Drug Monitoring Program mandate that prescriptions of controlled substances to be taken at home are logged centrally and that prescribers check before prescribing. This should be extended to ketamine, including in-clinic administration. The growing use of oral ketamine, which increases potential risks of overuse and diversion, emphasises the need for closer surveillance. Indeed, the recent legalisation of telehealth consultations and postal supply of ketamine in the USA during COVID increased access but raised concerns regarding patient safety, given the lack of rigorous monitoring. Significant efforts are being made towards reformulating oral ketamine to manage its misuse potential,^16^ and ketamine formulations will be entering phase 3 trials. Therefore, guidelines need to focus on regulating the use of compounded oral or sublingual liquids, lozenges or capsules. Finally, owing to the limited long-term real-world dosing data for ketamine, a national registry would also allow for tracking of outcomes across different routes and doses, which could help to optimise treatment. Therefore, these registers are vital for ensuring optimal cost-effectiveness, patient safety and treatment efficacy. Voluntary registries, such as those used to improve service delivery of electroconvulsive therapy,^17^ offer an alternative to mandatory national registries. These registries could be expanded to include treatments such as ketamine and esketamine, providing a framework for data collection and a shared clinical registry, informing how care is being delivered.

As we work to broaden the availability of ketamine treatments to those with TRD, any framework that promotes its use in a manner that is safe, effective and equitable is a step in the right direction. Refining our guidelines and vigilant monitoring will be imperative as we further our understanding of both the benefits and the limitations associated with ketamine therapy.

## References

[1] Jelen LA, Stone JM. Ketamine for depression. Int Rev Psychiatry 2021: 33(3), 207–28. 33569971 10.1080/09540261.2020.1854194

[2] Marcantoni WS, Akoumba BS, Wassef M, Mayrand J, Lai H, Richard-Devantoy S, et al. A systematic review and meta-analysis of the efficacy of intravenous ketamine infusion for treatment resistant depression: January 2009 – January 2019. J Affect Disord 2020; 277: 831–41. 33065824 10.1016/j.jad.2020.09.007

[3] Jelen LA, Young AH, Stone JM. Ketamine: a tale of two enantiomers. J Psychopharmacol 2021; 35: 109–23. 33155503 10.1177/0269881120959644 PMC7859674

[4] Horowitz MA, Moncrieff J. Are we repeating mistakes of the past? A review of the evidence for esketamine. Br J Psychiatry 2021; 219: 614–7. 32456714 10.1192/bjp.2020.89

[5] Kasper S, Young AH, Vieta E, Goodwin G, Meyer-Lindenberg A. Letter to *BJPsych* in response to Horowitz and Moncrieff. Br J Psychiatry 2021; 219: 619–20. 10.1192/bjp.2021.161 35048821

[6] Sanacora G, Frye MA, McDonald W, Mathew SJ, Turner MS, Schatzberg AF, et al. A consensus statement on the use of ketamine in the treatment of mood disorders. JAMA Psychiatry 2017; 74(4): 399–405. 28249076 10.1001/jamapsychiatry.2017.0080

[7] National Institute for Health and Care Excellence. *Depression in Adults: Treatment and Management*. NICE, 2022 (https://www.nice.org.uk/guidance/ng222/chapter/Recommendations#treatment-for-a-new-episode-of-more-severe-depression). 35977056

[8] Cleare A, Pariante CM, Young AH, Anderson IM, Christmas D, Cowen PJ, et al. Evidence-based guidelines for treating depressive disorders with antidepressants: a revision of the 2008 British Association for Psychopharmacology guidelines. J Psychopharmacol 2015; 29(5): 459–525. 25969470 10.1177/0269881115581093

[9] Bauer M, Pfennig A, Severus E, Whybrow PC, Angst J, Möller HJ, et al. World Federation of Societies of Biological Psychiatry (WFSBP) guidelines for biological treatment of unipolar depressive disorders, part 1: update 2013 on the acute and continuation treatment of unipolar depressive disorders. World J Biol Psychiatry 2013; 14(5): 334. 23879318 10.3109/15622975.2013.804195

[10] Kennedy SH, Lam RW, McIntyre RS, Tourjman SV, Bhat V, Blier P, et al. Canadian Network for Mood and Anxiety Treatments (CANMAT) 2016 clinical guidelines for the management of adults with major depressive disorder. Can J Psychiatry 2016; 61(9): 540. 27486148 10.1177/0706743716659417 PMC4994790

[11] McIntyre RS, Rosenblat JD, Nemeroff CB, Sanacora G, Murrough JW, Berk M, et al. Synthesizing the evidence for ketamine and esketamine in treatment-resistant depression: an international expert opinion on the available evidence and implementation. Am J Psychiatry 2021; 178(5): 383. 33726522 10.1176/appi.ajp.2020.20081251 PMC9635017

[12] Wajs E, Aluisio L, Holder R, Daly EJ, Lane R, Lim P, et al. Esketamine nasal spray plus oral antidepressant in patients with treatment-resistant depression: assessment of long-term safety in a phase 3, open-label study (SUSTAIN-2). J Clin Psychiatry 2020; 81(3): 19m12891. 10.4088/JCP.19m12891 32316080

[13] Short B, Dong V, Gálvez V, Vulovic V, Martin D, Bayes AJ, et al. Development of the Ketamine Side Effect Tool (KSET). J Affect Disord 2020; 266: 615–20. 32056935 10.1016/j.jad.2020.01.120 PMC7693479

[14] Swainson J, Klassen LJ, Brennan S, Chokka P, Katzman MA, Tanguay RL, et al. Non-parenteral ketamine for depression: a practical discussion on addiction potential and recommendations for judicious prescribing. CNS Drugs 2022; 36(3): 239–51. 35165841 10.1007/s40263-022-00897-2 PMC8853036

[15] Beaglehole B, Glue P, Clarke M, Porter R. Multidisciplinary development of ketamine guidelines for use by adult specialist mental health services in New Zealand. BJPsych Open 2023; 9(6): e191. 37828915 10.1192/bjo.2023.577 PMC10594164

[16] Glue P, Loo C, Fam J, Lane H, Young AH, Surman P. Randomized placebo-controlled phase 2 study of extended-release ketamine tablets (R-107) for treatment-resistant depression – the BEDROC study. *Res Sq* [Preprint ahead of publication] 31 Oct 2023. Available from: 10.21203/rs.3.rs-3501826/v1.

[17] Martin DM, Gálvez V, Lauf S, Dong V, Baily SA, Cardoner N, et al. The Clinical Alliance and Research in Electroconvulsive Therapy Network: an Australian initiative for improving service delivery of electroconvulsive therapy. J ECT 2018; 34(1): 7–13. 28658011 10.1097/YCT.0000000000000435

